# Tensile properties of a boron/nitrogen-doped carbon nanotube–graphene hybrid structure

**DOI:** 10.3762/bjnano.5.37

**Published:** 2014-03-20

**Authors:** Kang Xia, Haifei Zhan, Ye Wei, Yuantong Gu

**Affiliations:** 1 School of Chemistry, Physics and Mechanical Engineering, Queensland University of Technology, Brisbane QLD 4001, Australia

**Keywords:** doping, graphene, molecular dynamics simulation, nanotubes, tension, Young’s modulus

## Abstract

Doping is an effective approach that allows for the intrinsic modification of the electrical and chemical properties of nanomaterials. Recently, a graphene and carbon nanotube hybrid structure (GNHS) has been reported, which extends the excellent properties of carbon-based materials to three dimensions. In this paper, we carried out a first-time investigation on the tensile properties of the hybrid structures with different dopants. It is found that with the presence of dopants, the hybrid structures usually exhibit lower yield strength, Young’s modulus, and earlier yielding compared to that of a pristine hybrid structure. For dopant concentrations below 2.5% no significant reduction of Young’s modulus or yield strength could be observed. For all considered samples, the failure is found to initiate at the region where the nanotubes and graphene sheets are connected. After failure, monatomic chains are normally observed around the failure region. Dangling graphene layers without the separation of a residual CNT wall are found to adhere to each other after failure with a distance of about 3.4 Å. This study provides a fundamental understanding of the tensile properties of the doped graphene–nanotube hybrid structures, which will benefit the design and also the applications of graphene-based hybrid materials.

## Introduction

In recent years, low-dimensional structures such as carbon nanotubes (CNT) and graphene have attracted huge attention of the scientific community, because of their excellent performance in the fields of mechanics, photology, electronics and bio-sensing [1–2]. Through the chemical vapor deposition (CVD) method, a graphene–nanotube hybrid structure (GNHS) has been synthesized recently [3–5], which evidently demonstrates an improved performance for the application as field emission device when compared to the previous CNT–bulk-metal structures [6]. The hybrid structure extends the excellent thermal and electrical conductivity of CNT (1D) and graphene (2D) into three dimensions [7], and shows appealing applications in solar cells [8]. Furthemore, according to Fan et al. [9], owing to the double layer configuration, the CNT–graphene hybrid structures are expected to have a better electrochemical performance, which indicates that the hybrid structure is a good candidate for the usage of electrodes in supercapacitors.

In order to accommodate for various applications, different approaches have been developed to tailor the properties of nanomaterials. Doping is one of such schemes and has been extensively used in synthesizing derivatives from carbon-based materials (e.g., fullerene, nanotubes and graphene) [10]. Boron and nitrogen, which have comparable atomic size with carbon atom and can form strong valence bonds with carbon atoms, are the most frequently used doping elements for carbon-based materials [11]. The presence of boron and nitrogen atom induce significant variations in the electronic structure of graphene layer, which was shown by changes in the Raman spectra [12–13]. According to Panchakarla et al. [14], the doping induces donors and/or acceptors states, which modify the G band (in Raman spectrum) and are essential in facilitating the application of graphene-based electronics. The N-doped graphene is reported by Wang et al. [15] to be also a good candidate for the application as fuel cell electrocatalyst, in field-effect transistors, and in lithium batteries. Thus, especially N-doped nanotube–graphene hybrid structures have been envisioned to have promising potential applications in the field of catalysis, gas storage and energy storage [16].

The majority of the current works that are conducted on graphene variations are focusing on the electrical and chemical properties. However, to facilitate the applications of nanomaterials, a comprehensive understanding of their mechanical properties/performance is crucial. By using molecular dynamics (MD) simulations, Bohayra et al. [10] conclude that the content of nitrogen atoms (up to 6%) has a negligible effect on the Young’s modulus of a nitrogen-doped graphene layer, while the presence of nitrogen substitutions reduces the layer strength significantly. Only a few works have been devoted to examine the impact of dopant atoms on the mechanical properties of graphene. Huge efforts are still lying ahead especially for the newly synthesized CNT–graphene hybrid structure. Therefore, in this work, we will examine the impact of different densities and species of dopants on the tensile properties of the GNHS. The emphasis will be placed on Young’s modulus, *E*, yield strength, *YS*, and yield strain, *YP*.

## Computational details

In order to acquire the influence of the dopants on the mechanical properties of GNHSs, the large-scale atomic/molecular massively parallel simulator (LAMMPS) [17] is utilized to carry out the MD simulations. The pristine GNHS model is constructed by two graphene sheets, with zigzag and armchair edges along the *x*- and *y*-axes, respectively. We establish the initial structure according to pervious simulation models [8,18–21], i.e., a specific cylindrical hole is made in the graphene sheet to fit the armchair (4,4)-CNT with a height of 13.8 Å. Basically, three groups of sample structures have been tested, which include GNHS with nitrogen dopant (GNHS-N), GNHS with boron dopant (GNHS-B), and GNHS with both nitrogen and boron dopants (GNHS-NB). Each group contains six doped samples with different percentages of dopants. All structures for the simulations have an identical size of 24.6 × 5.6 × 1.4 nm^3^. For the sake of convenience, the percentage of dopants is included in the model name, e.g., a sample name ‘GNHS-1.5%N1.5%B means that the hybrid structure contains 1.5% of boron and nitrogen, respectively. The dopants are randomly distributed along the whole structure domain.

Similar to the work of Wei et al. [22], the C–C interatomic interactions are described by the commonly used empirical bond order (REBO) potential [23], which has been shown to represent the binding energy and elastic properties of graphene and CNT well [24]. Basically, the REBO potential is given as

[1]
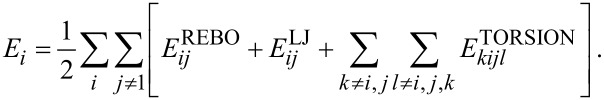


Here, the first term represents the interaction between *i* and *j* atoms, which strongly depends on the coordination. The second term accounts for a longer-ranged interaction that is depicted by a Lennard-Jones (LJ) potential, while the last term represents an explicit 4-body potential that describes various preferences for dihedral angles in hydrocarbon configurations. A Tersoff potential [25] is adopted to describe the atomic interactions of C–B, C–N and B–N. The N–N bond is considered to be chemically unstable. Thus, two adjacent N atoms are avoided in the model. It must be noted that the cut-off distance for the C–C bond has been modified from 1.7 Å to 2.0 Å according to a previous work [24]. Several studies have already demonstrated that a cut-off distance of 1.7 Å for carbon materials, which was used previously, will produce a spuriously high tensile force and lead to a nonlinear stress–strain curve [26–27]. In addition, the samples with higher densities of dopants contain all the dopant positions of the samples with lower doping percentages to ensure a reasonable comparison. To calculate the stress, the tensile force has been tracked. To lower the computational cost, the GNHS has been assumed as a continuum material, i.e., the cross-sectional area is a product of the width and height. Since we emphasize on the relative mechanical properties (Young’s modulus, yield strength) such an approximation will make no difference for the discussion.

**Figure 1:**
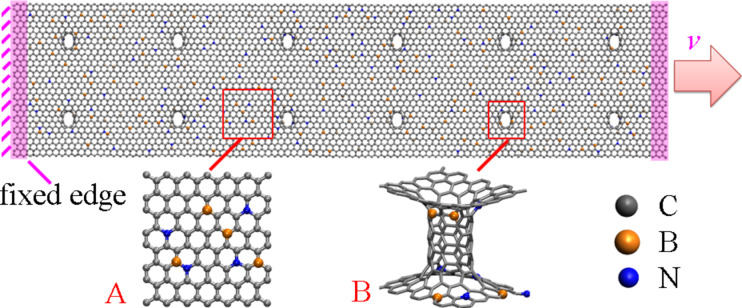
Schematic view of the model GNHS-2.0%N2.0%B. Inset ‘A’ shows the boron and nitrogen atoms located at the graphene layer, inset ‘B’ shows the boron and nitrogen atoms located at the connecting CNT.

At the beginning of the simulation, the conjugate gradient algorithm was applied to relax the model to a minimum energy state. We then used the Nose–Hoover thermostat [28–29] to equilibrate the GNHS at 1 K (NVT ensemble) for 500 ps at a time step of 1 fs. The extremely low temperature was chosen to exclude the thermal fluctuation influence. Figure 1 illustrates the atomic configuration of the GNHS-2.0N2.0B model and the simulation setup. A constant velocity of 0.005 Å/ps was applied to one end of the GNHS to exert the axial load (along the longitudinal *y*-axis), while the other end was held fixed. The equations of motion are integrated over time using a velocity-Verlet algorithm [30]. No periodic boundary conditions have been applied. The system temperature was maintained at 1 K during the simulation.

## Results and Discussion

**Figure 2:**
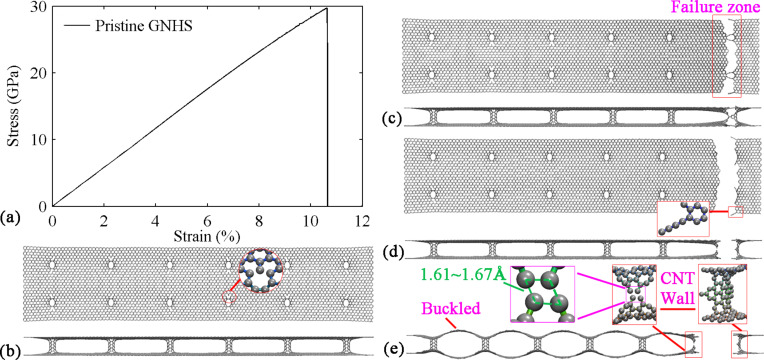
Simulation results for pristine GNHS: (a) Stress–strain curve; atomic configurations at the strain of (b) 0.085, inset shows the broken bonds around the connecting area; (c) 0.106; (d) 0.106, inset shows the monatomic chain; (e) 0.107.

Figure 2b and Figure 2c present the atomic configurations of the pristine hybrid structure before and after fracture. It is obvious that during the period of elastic deformation, all the C–C bonds have been stretched in the loading direction. With the increase of strain, the failure initiates from the region where the nanotubes and graphene sheets are connected. After the bonds begin to break, the hybrid structure quickly fails. This phenomenon is indicated by the sharp decrease of the stress (Figure 2a), which indicates a brittle behavior. Such brittle behavior can be easily explained as the tension loading direction is perpendicular to the axial direction of the CNT. Therefore, the tensile behavior of the GNHS is dominated by the graphene layer rather than by the nanotube and results in a brittle behavior.

During the failure of the structure, several short monatomic chains are formed at the front of the failure region (see inset in Figure 2d). The initial C–C bond length in graphene and CNT is 0.142 nm and is stretched to about 0.160 nm before breaking. It is observed from Figure 2e, that the two separated parts exhibit a bulked configuration eventually after the fracture of the hybrid structure. Strikingly, the upper and lower graphene layers (in the failure zone) are still separated by residual CNT walls. We notice that the length of the elongated C–C bonds in the left region (inset of Figure 2e) ranges from 1.61 to 1.67 Å, which is much longer than the typical length.

### Hybrid structures doped with nitrogen

**Figure 3:**
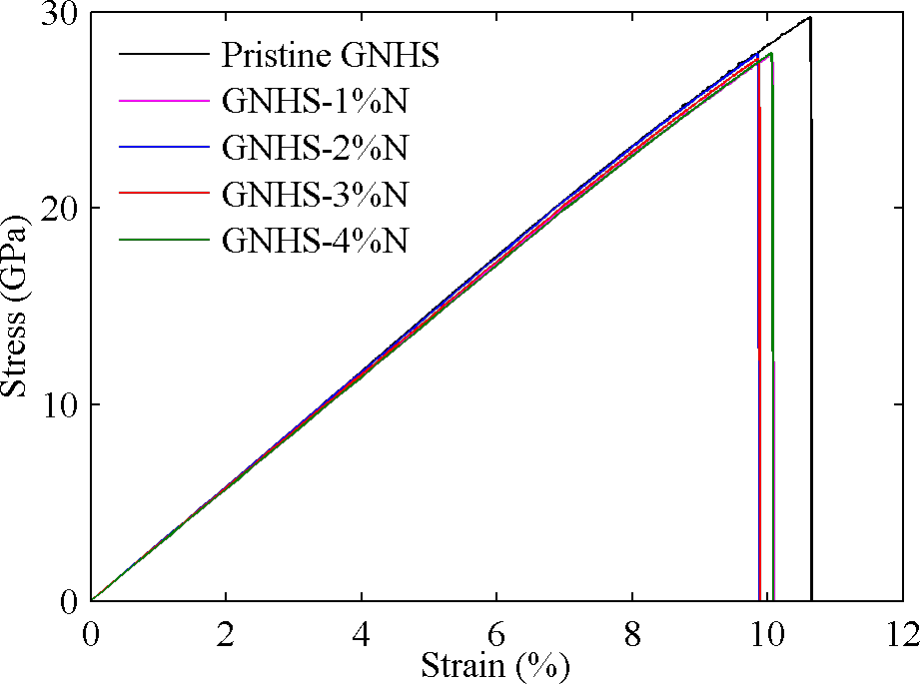
Stress–strain curves of GNHS with different percentage of N-dopants between 1% and 4%.

We then evaluate the tensile properties of doped GNHSs with different percentages of dopants. A concentration range of the N-dopants from 0.5% to 4.0% is considered. Figure 3 presents the stress–strain curves obtained from MD simulations. Similar to the pristine GNHS case, all N-doped GNHSs exhibit a linear stress–strain curve during the whole elastic deformation, and they nearly overlap at low strains (up to 4%). This phenomenon indicates that the Young’s moduli are only insignificantly changed. However as shown in Figure 3, yield strength, *YS*, and yield strain, *YP*, experience an apparent degradation. An increase of the dopant concentration, however, does not further reduce *YS* and *YP*. It is interesting to mention that an earlier work reported that 2% of N-doping in graphene monolayers induce a reduction of *YS* of more than 35% [10], which is much more significant than the reduction observed in the hybrid structures that are studied here. In addition, all stress–strain curves presented in Figure 3 show a sharp decrease of stress, which indicates a brittle behavior of the different GNHSs.

**Figure 4:**
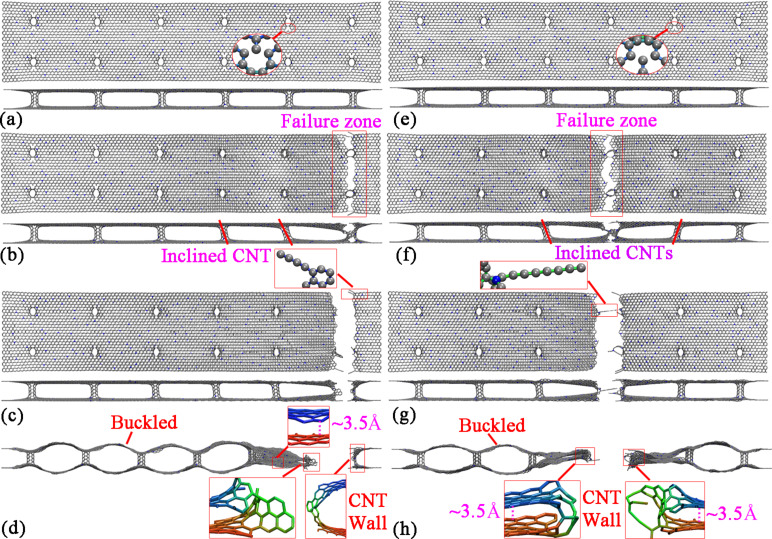
Atomic configurations of GNHS-2%N at a strain of: (a) 0.097; (b) 0.098; (c) 0.099, inset highlights the monatomic chain after the breaking of bonds; (d) 0.101, inset shows the dangling graphene layers. Atomic configurations of GNHS-3%N at the strain of: (e) 0.097; (f) 0.098; (g) 0.099; (h) 0.101.

In general, the GNHS with different densities of N-dopants behave similar to the pristine structure. It is found that the GNHS with 0.5%, 1.0%, 1.5%, 2.0%, 2.5% and 3.5% of N- dopants fracture at either the right or the left end of the structure. The atomic configurations of the GNHS with 2% of N-dopants are presented in Figure 4a-d. Before the initiation of failure, a shearing of the CNTs and an elongation of bonds are observed. Similar to the pristine GNHS case, failures start around the connection region (Figure 4a), and are followed by the formation of monatomic chains (Figure 4c). In Figure 4d, the buckled shape is formed because of the stress release after failure. Specifically, after failure, one end of the dangling graphene layers (left in Figure 4d) is separated by the residual CNT wall, and the other end exhibits self-adhesive behavior. Different from these cases, the other two structures with 3% and 4% N-dopant exhibit a fracture region around the middle of the hybrid structure, and the self-adhesive behavior is observed on both sides of the dangling graphene layers (shown in Figure 4h). Particularly, a longer chain is found that contains eight carbon atoms.

### Hybrid structures doped with boron

**Figure 5:**
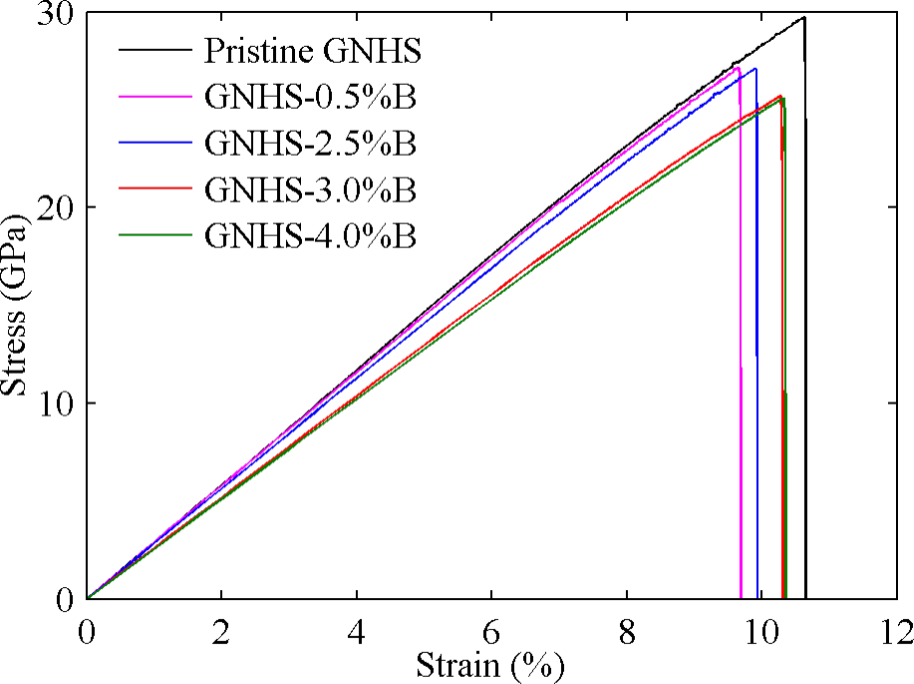
Stress–strain curves of GNHS with different percentage of B-dopant ranging from 0.5% to 4%.

Besides nitrogen, boron is another common doping element. Thus, we continue our investigation by considering the GNHS with different percentages of B-dopants. Similar to the cases of nitrogen doping, an evident decrease of the yield strength and early yielding are observed (Figure 5). Within the elastic deformation region, the increase of dopant leads to a marginal shift to the slope of the stress–strain curve, which indicates an insignificant reduction in Young’s modulus. Of all samples studied, the one with 0.5% B-dopant exhibits the highest Young’s modulus and *YS*, which are 0.290 TPa and 27.13 GPa, respectively. While the case with 4% B-dopant shows the lowest Young’s modulus and *YS*. Importantly, we found that *YS* is not reduced linearly with increasing boron percentage.

**Figure 6:**
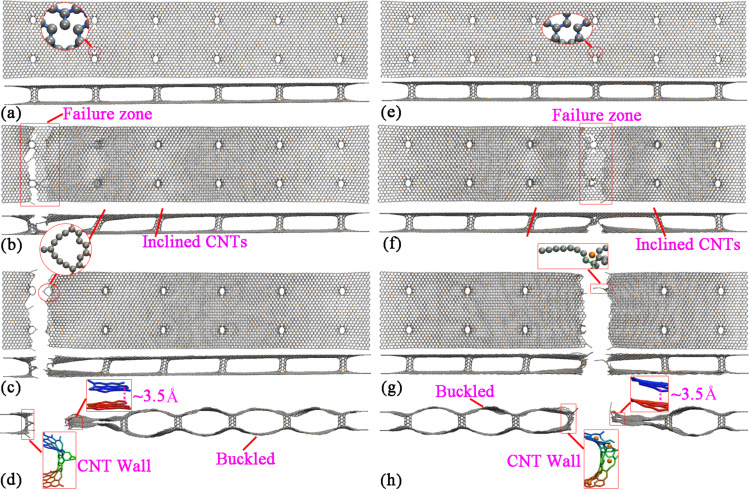
Atomic configurations of GNHS-2.5%B at the strain of: (a) 0.094; (b) 0.102; (c) 0.103, inset reveals the formation of a monatomic ring; (d) 0.104. Atomic configurations of GNHS-3%B at the strain of: (e) 0.099; (f) 0.106; (g) 0.107; (h) 0.108.

Regarding the deformation process the hybrid structures with 0.5%, 1.0%, 1.5%, 2.0% and 2.5% B-dopant share a similar pattern. Specifically, Figure 6a–d illustrate the atomic configurations of GNHS-2.5%B at different strains. As in the previously considered cases, the failure initiates around the connection region and monatomic chains are formed (highlighted in Figure 6c). Interestingly, these monatomic chains have formed three rings around the failure region. After failure, a buckled shaped is formed, and one end the dangling graphene layers are separated by the residual CNT wall, while the other end shows self-adhesive behavior. Besides, in the other three cases (with 3.0%, 3.5% and 4.0% B-dopant) the fracture is observed around the middle area.

### Hybrid structures doped with nitrogen and boron

**Figure 7:**
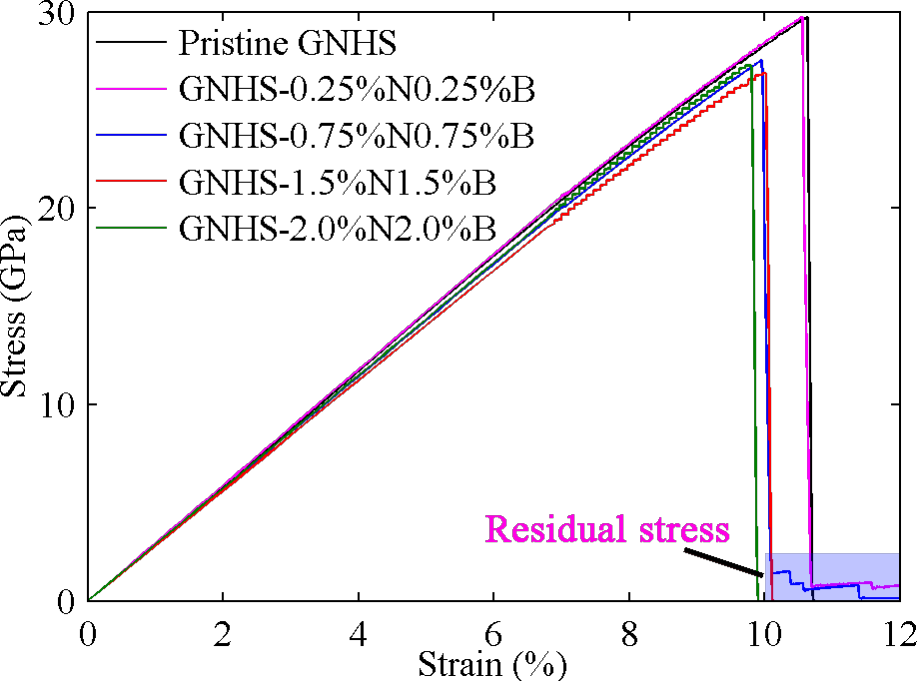
Stress–strain curves of GNHS with different densities of B- and N-dopant.

In order to improve the ferroelectric properties and the layer resistivity, N and B doping is widely adopted in thin films studies [31–34]. In this section, we consider a hybrid structure that is doped with both nitrogen and boron. The stress–strain curve is presented in Figure 7. As can be seen, GNHS-0.25%N0.25%B has similar *YS* and *YP* as the pristine GNHS, which are 29.27 GPa and 10.55%, respectively. With an increase of the percentage of nitrogen and boron dopants to 0.75%, a considerable drop in *YS* and *YP* is observed (see Figure 7). It is worth to mention that, for GNHS-0.25%N0.25%B and GNHS-075%N0.75%B, the stress does not decrease directly to zero after fracture. An explanation for this phenomenon is given below.

**Figure 8:**
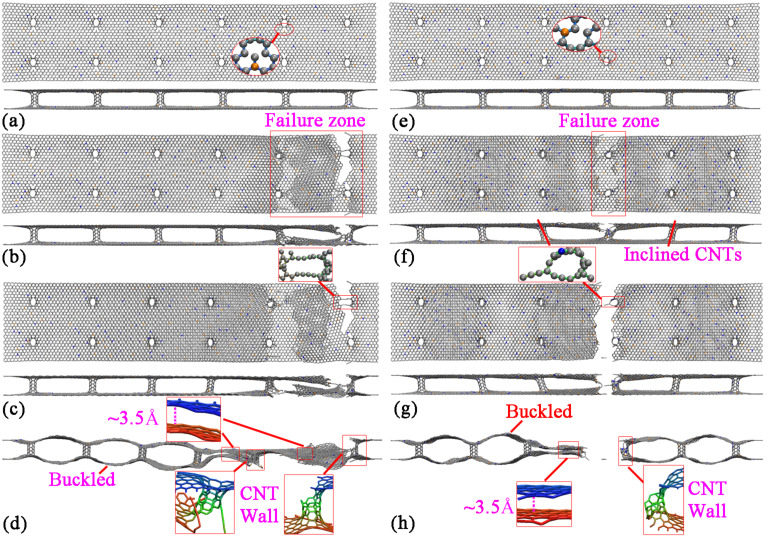
Atomic configurations of GNHS-0.75%N0.75%B at the strain of: (a) 0.097; (b) 0.101 (c) 0.102; (d) 0.115. Atomic configurations of GNHS-1.5%N1.5%B at the strain of: (e) 0.097; (f) 0.101; (g) 0.102; (h) 0.103.

Besides of the failure around the end of the structure, fractures at other locations are also observed for the hybrid structure with both B- and N-dopants. Figure 8a–d illustrate the atomic configurations of the case with 0.75%B and 0.75%N at different strains. Surprisingly, the hybrid structure is found to fracture around four CNTs. After failure, the upper layer is found to break at the outermost two CNTs at the right end, while the lower layer fractures at the second outermost two CNTs. Such deformation is found to result two dangling layers (upper and lower) that adhere to each other. This adhesive behavior is the reason for the residual stress, which is highlighted in Figure 7. With sufficient elongation, the dangling layers finally separate from each other by van der Waals interaction. The failure of the hybrid structure around the middle region is also witnessed. As shown in Figure 8f, the top and bottom layers of GNHS-1.5%N1.5%B fracture simultaneously around the two connecting CNTs. In all investigated cases, the self-adhesive behavior between the dangling layers and the bulked configuration of the structure is observed after failure. It is necessary to point out that, the boundary condition applied in this work is non-periodic. According to the results presented in Figure 4, Figure 6 and Figure 8, the location of the fracture region is quite random during the simulation. According to previous work on metal nanowires [35] the location of necking is highly related to the strain rate, which could be predicted by the longitudinal wave propagation equation. However, the difference to a nanowire is that the hybrid structure is intrinsically inhomogeneous. Such an inhomogeneity is believed to introduce a local concentration of stress around the connecting regions, and thus could lead to the phenomenon that the fracture always starts around one of these connecting areas.

**Figure 9:**
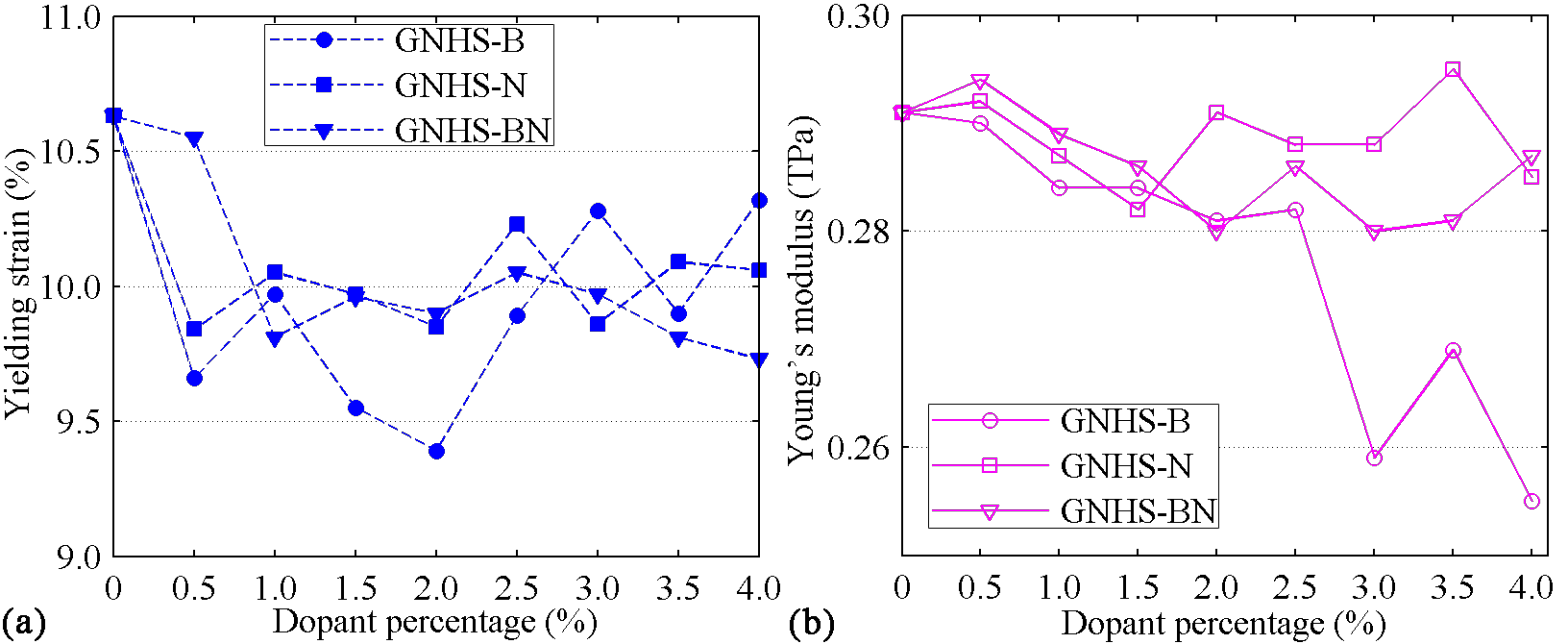
Yield strain, *YP*, and Young’s modulus, *E*, as a function of the concentration of N-, B-, and NB-dopants.

Before concluding, we compare the yield strains and Young’s moduli of all studied cases. Figure 9a shows the yield strain as a function of the concentration of the dopant. Clearly, the existence of different dopants reduces *YS*. However, the there is no strong correlation between the concentration of the dopant and the reduction of *YS*. For all types of dopants, the reduction is found to fluctuate around 10% (Figure 9a). In most of the circumstances, the hybrid structures with dopants exhibit low Young’s moduli. However, for GNHS-0.5%N and GNHS-3.5%N, the Young’s modulus is even higher than that of the pristine GNHS, which are 0.292 TPa and 0.295 TPa, respectively. Figure 9b shows that increase of boron doping results in a sharp reduction of the Young’s modulus, while the other considered cases exhibit Young’s moduli around 0.29 TPa.

## Conclusion

Basing on the large-scale MD simulation, the tensile properties of a graphene–carbon nanotube hybrid structure with different dopants have been investigated. It is found that with the presence of dopants, the hybrid structures usually exhibit a lower yielding strength, Young’s modulus, and earlier yielding when compared to a pristine hybrid structure. Young’s modulus and yielding strength are not reduced when the concentration of dopants increases further. For all considered samples, the failure is found to initiate in the region where the nanotubes and graphene sheets are connected. After failure, monatomic chains are normally observed around the failure region. The dangling graphene layers are found to adhere to each other through van der Waals interactions with a distance of around 3.4 Å. This study provides a fundamental understanding of the tensile properties of the doped graphene–nanotube hybrid structures, which will benefit the design and also the applications of graphene-based hybrid materials.
